# Board of Pension Examining Surgeons

**Published:** 1871-03

**Authors:** 


### Board of Pension Examining Surgeons.

The following declaration sufficiently explains itself. It commends itself to every thinking mind:

Office of the Board of Pension Examining Surgeons,
For the 28th Congressional District of New York,
Rochester, February 27th, 1871.

Hon. H. Van Aernam, Commissioner of Pensions:

Sir,—We, the undersigned, composing the Board of Pension Examining Surgeons for the 28th Congressional District of New York, have seen with much surprise the charge of proscription made against you because of your recent action in organizing Boards of Examiners.

It is difficult to understand how this charge can be made by any one conversant with the practical operations of the Bureau.

Since the adoption of the system of Boards has superceded single examining surgeons, harmony and unity of action, always desirable, have become indispensible. We are required to examine, consult together, and determine the nature and permanence of injuries and maladies, and the probability of their cure. The various "schools" you mention hold such diverse and opposite opinions that they cannot work together towards uniform results. Maladies which one considers incurable, can be controlled and removed by the minutest remedies. So incompatible are the different systems, that the Supreme Court of this State has decided that a person who offers to practice Homoeopathy or Allopathy, as his patients may wish, is "practically a quack in his proiession." (*Ex parte Paine*, 1 *Hill*, 665.) And yet it is claimed that the Bureau of Pensions should recognize all systems alike.

The duty is imposed upon you of selecting such agents as will, in your opinion, most accurately define the condition of each claimant, and so determine the amount of his pension. In making this selection you must necessarily choose such individuals as will most effectually promote the interests both of the Government and of the Pensioners, and secure equal and uniform justice throughout the whole United States.

The end could not be accomplished by organizing Boards belonging to the different "Schools" you mention. The result would be that a pensioner certified by one Board would receive the smallest allowance provided by law for a temporary malady, while another pensioner, in precisely the same condition, certified by a Board of a different stamp, would receive the largest pension awarded for a permanent and incurable disability. As well might a Commission of Jews, Christians Turks and Infidels sit in judgment upon alleged departures from the True Faith!

Whatever system or school may be deemed best to the interest of the Government and of the Pensioners, you are in duty bound to adopt, and that system should be uniform throughout the whole country. The selection of individuals deemed most fit for the office must necessarily involve a choice among the schools to which they belong. All systems are not equally good. You are bound to select that which, in *your judgment*, is best, and you are thus compelled to give the preference to one over all the rest.

Very Respectfully,

Your Obedient Servants,

B. L. Hovey, Pres't of Board.
[Signed] H. F. Montgomery, Treasurer.
D. Little, Secretary.

We, the subscribers, residing in the city of Rochester, and in the 28th Congressional District of New York, fully concur in the opinions expressed in the above letter, and believe that the principles stated are absolutely necessary to secure an uniform Pension system throughout the United States.

(Signed by the Physicians of the district.)

